# O7-5 School-based interventions to reduce accelerometer based children's sedentary time: presentation of a systematic review

**DOI:** 10.1093/eurpub/ckac094.053

**Published:** 2022-08-29

**Authors:** Caroline Bernal, Lena Lhuisset, Nicolas Fabre, Julien Bois

**Affiliations:** STAPS, Université de Pau et des Pays de l'Adour (UPPA), Tarbes, France

**Keywords:** sedentary time, children, school, health, accelerometry

## Abstract

**Background:**

Sedentary time (ST) is considered as a major public health concern. Children are particularly vulnerable as their ST increase with age. Therefore, school-based interventions aimed to reduce and prevent children ST are burgeoning. Previously a review found that school-based interventions proposing multiples components which include standing desks appeared to be more effective compared to uni-component studies (Hegarty et al., 2016). However, this result only depended on 11 records published before 2016 and must be verified since the growing number of school-based interventions are proposed.

Objective: To continue the evaluation of the effectiveness of school-based interventions published since the previous review (2016).

**Methods:**

A total of 4 databases were examined: PubMed-Medline, PsycINFO, ScienceDirect and Google Scholar. The search was conducted using keywords in English, the main ones being: 1) “Sedentary Time” 2) “Intervention” 3) “Child”, 4) “School”. Records published between August 2016 and August 2019 with objective measure of ST were analysed. Data were collected and compiled by an author according to PRISMA criteria.

**Results:**

14 studies were included: 9 studies were multi-components (64%) and 5 were uni-component (36%). The method of measurement used in all studies was accelerometry. Environmental and organizational changes were the most used components. Among fourteen, nine studies reported significant results on post-intervention: respectively, with three being uni-component and six being multi-components. The long-term effect remain undetermined.

**Conclusion:**

Uni component studies used only environmental reorganization which reduces ST in the short term. This finding had already been supported in previous interventions studies using standing desks that had been identified by the last systematic review (Aminian et al., 2015; Clemes et al., 2016). The uni- and multi-components studies do not differ clearly in the results obtained. However, multi-components interventions seem to be the more promising strategy to reduce ST and to develop healthy habits at long term. Interventions with follow-up measures at long term are required.

